# Corneal Stromal Cell Growth on Gelatin/Chondroitin Sulfate Scaffolds Modified at Different NHS/EDC Molar Ratios

**DOI:** 10.3390/ijms14012036

**Published:** 2013-01-21

**Authors:** Jui-Yang Lai

**Affiliations:** 1 Institute of Biochemical and Biomedical Engineering, Chang Gung University, Taoyuan 33302, Taiwan; E-Mail: jylai@mail.cgu.edu.tw; Tel.: +886-3-211-8800 (ext. 3598); Fax: +886-3-211-8668; 2 Biomedical Engineering Research Center, Chang Gung University, Taoyuan 33302, Taiwan; 3 Molecular Medicine Research Center, Chang Gung University, Taoyuan 33302, Taiwan

**Keywords:** gelatin, chondroitin sulfate, carbodiimide chemistry, scaffold, corneal keratocyte

## Abstract

A nanoscale modification strategy that can incorporate chondroitin sulfate (CS) into the cross-linked porous gelatin materials has previously been proposed to give superior performance for designed corneal keratocyte scaffolds. The purpose of this work was to further investigate the influence of carbodiimide chemistry on the characteristics and biofunctionalities of gelatin/CS scaffolds treated with varying *N*-hydroxysuccinimide (NHS)/1-ethyl-3-(3-dimethyl aminopropyl) carbodiimide hydrochloride (EDC) molar ratios (0–1) at a constant EDC concentration of 10 mM. Results of Fourier transform infrared spectroscopy and dimethylmethylene blue assays consistently indicated that when the NHS to EDC molar ratio exceeds a critical level (*i.e*., 0.5), the efficiency of carbodiimide-mediated biomaterial modification is significantly reduced. With the optimum NHS/EDC molar ratio of 0.5, chemical treatment could achieve relatively high CS content in the gelatin scaffolds, thereby enhancing the water content, glucose permeation, and fibronectin adsorption. Live/Dead assays and interleukin-6 mRNA expression analyses demonstrated that all the test samples have good cytocompatibility without causing toxicity and inflammation. In the molar ratio range of NHS to EDC from 0 to 0.5, the cell adhesion ratio and proliferation activity on the chemically modified samples significantly increased, which is attributed to the increasing CS content. Additionally, the materials with highest CS content (0.143 ± 0.007 nmol/10 mg scaffold) showed the greatest stimulatory effect on the biosynthetic activity of cultivated keratocytes. These findings suggest that a positive correlation is noticed between the NHS to EDC molar ratio and the CS content in the biopolymer matrices, thereby greatly affecting the corneal stromal cell growth.

## 1. Introduction

In the field of tissue engineering, the design of biomaterial scaffolds is always an important subject [1]. A number of scaffold materials derived from either natural or synthetic origin can be used to produce an extracellular matrix (ECM) template for cell growth and organization. Over the past few years, tissue engineering has emerged as a promising new technology to overcome the common limitations of corneal replacement with an allograft [2]. Griffith *et al.* have introduced the hydrated collagen and *N*-isopropylacrylamide copolymer-based hydrogels containing laminin adhesion pentapeptide motif to promote epithelial stratification and neurite in-growth during corneal reconstruction [3]. Later, the group reports work on the development of a simple, cross-linked collagen tissue substitute for potential clinical application given that the human corneal matrices comprise mainly type I collagen and glycosaminoglycans (GAGs) [4]. Porcine collagen cross-linked with 1-ethyl-3-(3-dimethyl aminopropyl) carbodiimide hydrochloride (EDC)/*N*-hydroxysuccinimide (NHS) demonstrates stable graft-host integration following implantation in an animal model. In another study, Vrana *et al.* fabricate the collagen-chondroitin sulfate (CS) scaffolds to reconstruct full-depth corneas *in vitro* using normal human keratocytes, adult limbal stem cell-derived human epithelial cells, and transformed human endothelial cells [5].

In order to improve the stability of collagenous materials, carbodiimide chemistry is one of the most widely used cross-linking methods to create intermolecular covalent bonds between biopolymer chains. In our laboratory, this chemical cross-linker has been applied to the treatment of various ocular cell sheet carriers made from biomaterials such as hyaluronic acid [6], gelatin [7], and amniotic membrane [8]. According to the general reaction mechanism [9], *N*-substituted carbodiimide can activate the carboxyl groups of biomacromolecules to generate a highly reactive *O*-acylisourea intermediate. The formation of cross-links within the proteinaceous matrices is achieved by nucleophilic attack of free amine residues on the activated carboxyl residues, with the release of water-soluble urea by-product that exhibits relatively low cytotoxicity. Therefore, EDC-mediated conjugation is also popular for biomaterial processing. More recently, to fabricate biodegradable *in situ* forming delivery systems for intracameral administration of antiglaucoma medications, the aminated gelatin was grafted with carboxylic end-capped poly(*N*-isopropylacrylamide) by means of carbodiimide chemistry [10]. On the other hand, to develop corneal keratocyte scaffolds, the porous gelatin template was modified with CS by direct linking of the EDC-activated carboxylic acid groups of the polysaccharide molecules to the protein molecules [11]. In comparison with collagen, gelatin is cheaper and has relatively lower antigenicity while this material still contains cell adhesion sequences (RGD) for tissue engineering applications [12].

Although carbodiimide chemistry scheme is simple, the occurrence of hydrolysis of a highly reactive *O*-acylisourea intermediate is noticed. The NHS is known to be a promoter of EDC-based reaction. It is usually added to the solution mixture to give the NHS-activated carboxylic acid groups, which prevents the side reaction of hydrolysis of *O*-acylisourea [8]. However, investigators have reported that during chemical modification of gelatin matrices, the reaction of NHS with carbodiimide may compete with the cross-linking reaction [13]. A study from Olde Damink *et al.* has also indicated that the highest values of shrinkage temperature and lowest values of the free amine group content are found for the dermal sheep collagen samples cross-linked with a NHS/EDC molar ratio of 0.4 [14]. These phenomena have motivated us to investigate the relationship between the amount of NHS and the EDC-mediated biomaterial modification for the preparation of corneal keratocyte scaffolds.

As an ophthalmic biomaterial, gelatin is derived from denaturation of collagen and displays excellent biocompatibility [15]. For corneal regenerative medicine, the gelatin materials have been used as cell delivery systems to maintain tissue-like architecture of sheet transplants and facilitate a minimally invasive surgical approach [16,17]. On the other hand, as one of the main components of the ECM, CS is a linear anionic polysaccharide composed of repeating disaccharide units of d-glucuronic acid and *N*-acetyl-d-galactosamine. Due to its essential role in many crucial biological functions and cellular processes, CS has been extensively used as a cell scaffold material for tissue engineering [18]. We previously proposed a nanoscale modification strategy that could optimally incorporate biomolecule to give superior performance for designed corneal scaffolds [11]. The porous gelatin membrane scaffolds were immersed in an ethanol/water mixture containing 10 mM EDC, 2 mM NHS, and 0%–1.25% (*w*/*v*) CS for chemical modification. Although the treatment with 1.25% CS led to the covalent incorporation of larger amounts of CS into the porous gelatin samples, the relatively high hydration levels of materials restricted their application as corneal stromal cell scaffolds. Therefore, the concentration of 0.25% CS was selected for subsequent work. It is generally believed that the design of a suitable scaffold is multi-faceted and involves several sectors. As mentioned above, NHS/EDC molar ratio is an important factor that is taken into consideration when studying biomaterial modification. The purpose of this work was to further examine the effect of carbodiimide chemistry on the characteristics and biofunctionalities of gelatin/CS scaffolds. The porous biopolymer matrices modified at varying NHS/EDC molar ratios were evaluated by determinations of their CS content, water absorbing capacity, Young’s modulus, nutrient permeability, cytocompatibility, and protein adsorption capacity. In particular, the role of NHS to EDC molar ratio in constructing biomaterial scaffolds for the regulation of corneal stromal cell growth was studied *in vitro*, using cell adhesion and proliferation assays as well as ECM production analyses.

## 2. Results and Discussion

### 2.1. Fourier Transform Infrared (FTIR) Spectroscopy

**Figure 1:**
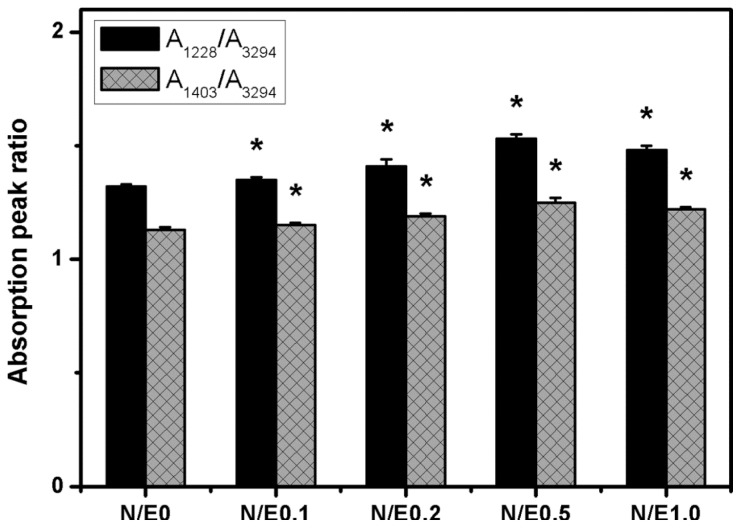
The absorption peak ratios of the S=O stretching to N–H stretching bands (A_1228_/A_3294_) and C–O stretching to N–H stretching bands (A_1403_/A_3294_) in Fourier transform infrared spectroscopy for the gelatin/chondroitin sulfate (CS) samples modified at different NHS/EDC molar ratios. An asterisk indicates statistically significant differences (*p < 0.05; *n* = 3) *vs.* N/E0 (compared only within *A*_1228_/*A*_3294_ or *A*_1403_/*A*_3294_ groups).

In the present work, the porous gelatin sheets were cross-linked with 50 mmol/L EDC and 10 mmol/L NHS. During the subsequent reaction with 0.25% (*w*/*v*) CS, the cross-linked biopolymer samples were again immersed in an ethanol/water mixture containing 10 mmol/L EDC and 0–10 mmol/L NHS. The pore size (~50 μm) and porosity (~25%) of various chemically modified scaffolds were similar to those found before treatment with CS. The results indicate that carbodiimide at a low concentration does not alter the porous structure of gelatin/CS scaffolds. Additionally, the cross-linking index of gelatin remained unchanged following repeated exposure to EDC and NHS in the absence of CS, suggesting that the NHS-activated carboxylic acid groups of glutamic or aspartic acid residues have already completely reacted with free amino groups of lysine residues to generate amide bonds in gelatin. By contrast, the decrease in the number of free amino groups of gelatin was observed after reaction with the NHS-activated carboxyl groups of mobile CS molecules. Representative spectra for the gelatin/CS scaffolds modified at different NHS/EDC molar ratios are shown in Figure S1. The infrared absorption peak ratios, measured with respect to the N–H band around 3300 cm^−1^, are summarized in Figure 1. The intensity ratios of *A*_1228_/*A*_3294_ and *A*_1403_/*A*_3294_ are indicative of CS signals. In the range of 0–0.5, both the intensity ratios significantly increased with increasing NHS/EDC molar ratio (*p* < 0.05). Our data demonstrate that the addition of larger amounts of NHS may enhance the efficiency of coupling of CS to gelatin chains. On the other hand, it has been reported that when high ratios of NHS to EDC are used for the treatment of collagen materials, the reaction between EDC and NHS leads to the depletion of EDC from the cross-linking solution [14]. Similar to their findings, the present results suggest that when the NHS to EDC molar ratio exceeds a critical level (*i.e*., 0.5), the efficiency of modification of gelatin scaffolds with CS is significantly reduced (*p* < 0.05).

### 2.2. Dimethylmethylene Blue Assays

**Figure 2:**
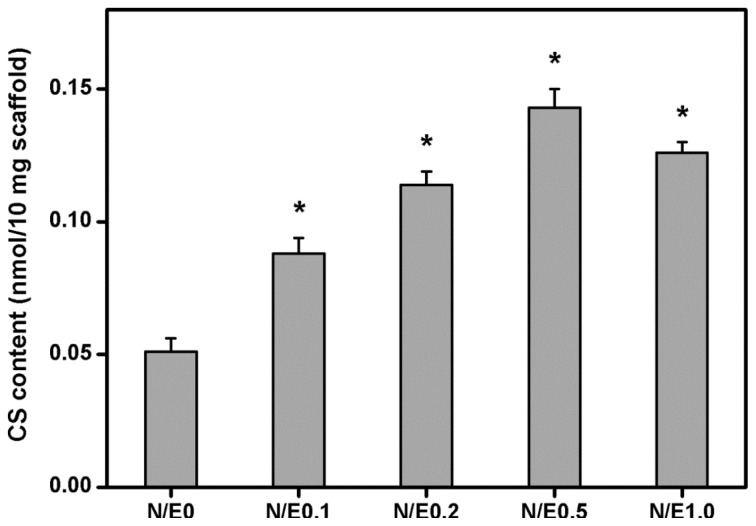
The CS content (determined by Dimethylmethylene blue (DMMB) assay) of the gelatin/CS scaffolds modified at different NHS/EDC molar ratios. An asterisk indicates statistically significant differences (*p < 0.05; *n* = 5) as compared to the N/E0 groups.

Figure 2 further shows the results of incorporation of CS into the gelatin scaffolds modified at different NHS/EDC molar ratios. In the N/E0 groups, the samples had the CS content of 0.051 ± 0.005 nmol/10 mg scaffold. It was significantly lower than those of the N/E0.1 (0.088 ± 0.006 nmol/10 mg scaffold), N/E0.2 (0.114 ± 0.005 nmol/10 mg scaffold), and N/E0.5 (0.143 ± 0.007 nmol/10 mg scaffold) groups (*p* < 0.05). In addition, the CS content was significantly reduced to 0.126 ± 0.004 nmol/10 mg scaffold in the N/E1.0 groups compared to that in the N/E0.5 groups (*p* < 0.05). Once again, a positive correlation was noticed between the NHS to EDC molar ratio and the CS content in the gelatin scaffold. We have previously performed a microscale modification of porous gelatin samples with CS [11]. However, the hydrophilicity and mechanical stability of the scaffold materials are much different from those of native corneas. Here, the nanoscale modification is reported by investigating the effect of carbodiimide chemistry on the functionality of gelatin/CS scaffolds and their interaction with corneal stromal cells.

### 2.3. Water Content Measurements

**Figure 3:**
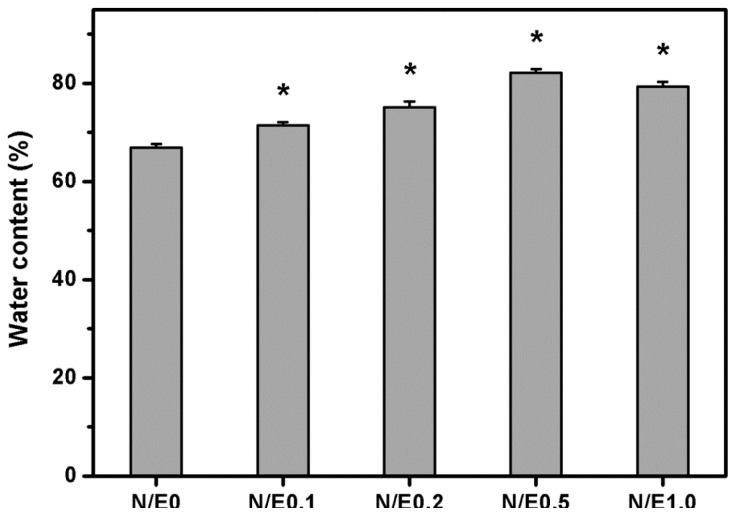
Equilibrium water content of the gelatin/CS scaffolds modified at different NHS/EDC molar ratios. An asterisk indicates statistically significant differences (*p < 0.05; *n* = 5) as compared to the N/E0 groups.

Figure 3 shows the water content of the gelatin/CS scaffolds modified at different NHS/EDC molar ratios. After immersion in BSS at 34 °C for 4 h, the samples from N/E0 groups had an equilibrium water content of 66.9% ± 0.7%, which was significantly lower than those of all the other groups (*p* < 0.05). This finding indicates the role of NHS in the regulation of hydrophilic properties of carbodiimide treated biopolymer scaffolds. In addition, the data for the samples modified with CS in the presence of varying amounts of NHS ranged from 71.4% to 82.1%. During the hydration of gelatin, the water molecules penetrate the tiny interstices of triple-helical fibrils in the matrix [7]. Since the CS is typically a hydrophilic polysaccharide, its incorporation into the scaffolds may further enhance the water absorbing capacity. A potential tissue-engineered cornea replacement needs to meet the requirements of the water content. Given that the normal cornea has a water content of around 76% [19], the gelatin samples modified with CS in the absence of NHS may be unsuitable for use as cell scaffold materials in corneal stromal tissue engineering. On the other hand, Monti *et al.* have demonstrated that the epithelial and/or endothelial damage may occur when the hydration levels of cornea increase up to 83%–92% [20]. Based on these earlier observations and our current results, the water content of all the gelatin/CS scaffolds is below the critical value, suggesting that even the treatment with CS at NHS/EDC molar ratio of 0.5, the gelatin materials possess acceptable hydrophilic properties.

### 2.4. Mechanical Tests

**Figure 4:**
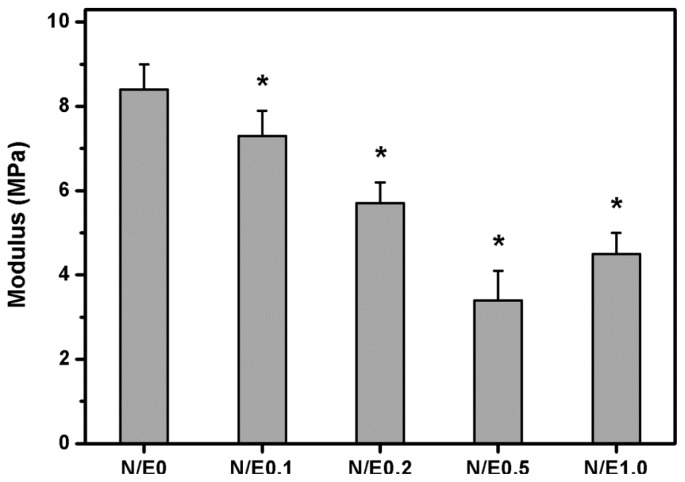
Young’s modulus of the gelatin/CS scaffolds modified at different NHS/EDC molar ratios. An asterisk indicates statistically significant differences (*p < 0.05; *n* = 9) as compared to the N/E0 groups.

In the present work, the mechanical properties of hydrated biopolymer matrices were examined since the scaffolds must continuously come into contact with the medium during *in vitro* RCK cultivation. Figure 4 shows the tensile test results for the gelatin/CS scaffolds modified at different NHS/EDC molar ratios. The Young’s modulus in the N/E0, N/E0.1, N/E0.2, N/E0.5, and N/E1.0 groups was 8.4 ± 0.6, 7.3 ± 0.6, 5.7 ± 0.5, 3.4 ± 0.7, and 4.5 ± 0.5 MPa, respectively. There were significant differences among these groups (*p* < 0.05). The results indicate that the mechanical stability of the scaffold materials is indirectly affected by the variation of the molar ratio of NHS to EDC. Our previous studies have shown that the increase in water absorption is usually accompanied by reduced structural strength of the ocular cell/tissue carriers made from either gelatin [21,22] or hyaluronic acid [23,24]. As mentioned, the chemically modified gelatin samples with more CS content are found to have higher water content. The alteration in water absorbing capacity may account for the observed differences in Young’s modulus in various scaffolds. These results support the report by Zeugolis *et al.* demonstrating that water content plays an important role in determining the mechanical properties of extruded collagenous materials [25]. Here, the Young’s modulus of the scaffold materials from all groups except the N/E0 group is acceptable because it corresponds to the range of the measured normal corneal tissue values (*i.e.*, 0.3–7 MPa) [26].

### 2.5. Glucose Permeation Studies

**Figure 5:**
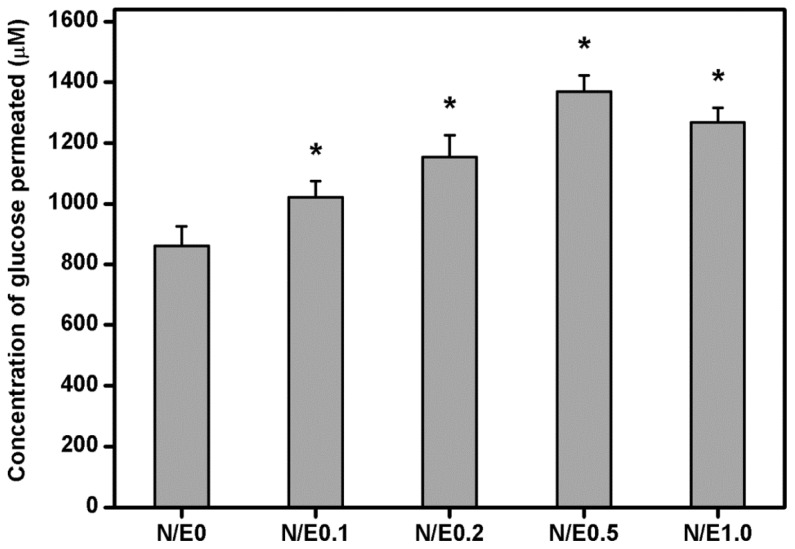
Concentration of glucose permeated through various gelatin/CS scaffolds at 34 °C. An asterisk indicates statistically significant differences (*p < 0.05; *n* = 5) as compared to the N/E0 groups.

Glucose is known to be one of the major nutrients for growth of the cornea. It has been documented that the glucose may permeate following the concentration gradient from the aqueous humor into the cornea [27]. In this study, the gelatin/CS scaffolds modified at different NHS/EDC molar ratios were evaluated for their ability to allow the permeation of glucose. Figure 5 shows the concentration of glucose in the receptor chamber after permeation through the gelatin/CS scaffolds at 34 °C for 12 h. Our results demonstrated that there were significant differences in the detected glucose concentration between N/E0 (861.6 ± 65.3 μM), N/E0.1 (1020.9 ± 52.7 μM), N/E0.2 (1153.5 ± 71.8 μM), N/E0.5 (1369.2 ± 53.1 μM), and N/E1.0 (1267.1 ± 48.5 μM) groups (*p* < 0.05). These findings indicate that the variation of amount of permeated nutrient is highly correlated with the CS content in the gelatin scaffold. Under physiological conditions (*i.e.*, pH 7.4), the carboxyl groups of CS are ionized and the carboxylate ions repel each other electrostatically. Hence, the increase in the CS content may cause an increase in the repulsive forces, thereby contributing to the expansion of the biopolymer networks and promoting the permeation of glucose through the gelatin/CS scaffolds.

### 2.6. *In Vitro* Biocompatibility Studies

**Figure 6:**
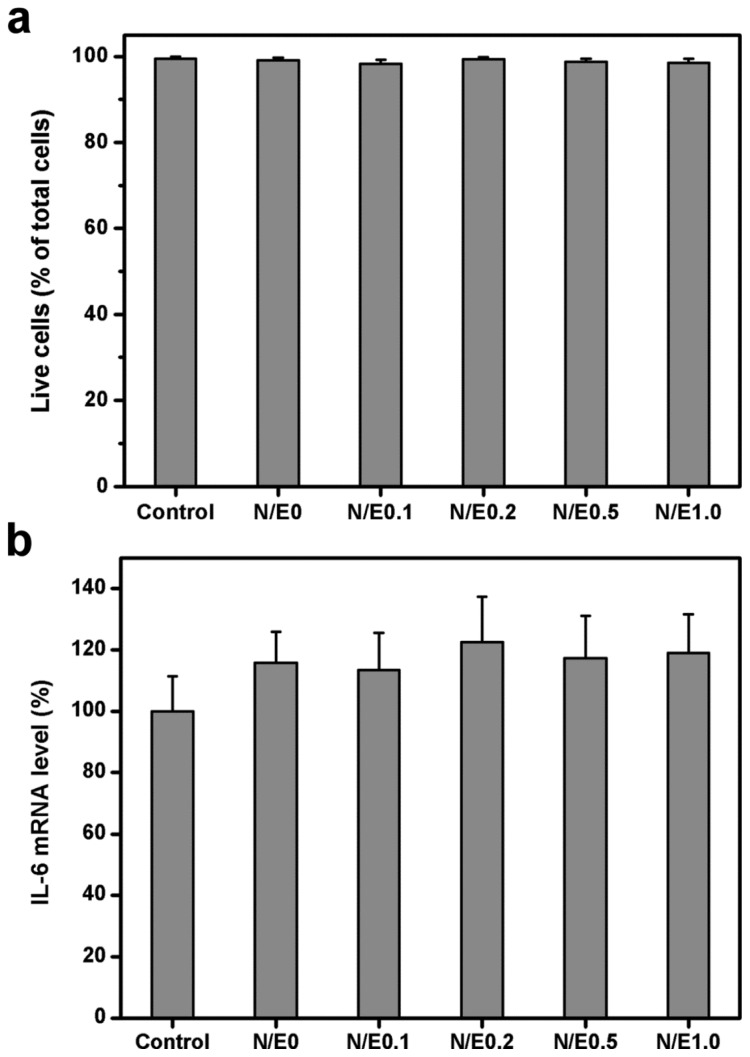
(**a**) Mean percentage of live cells in the rabbit corneal keratocyte (RCK) cultures as measured by the Live/Dead assay after a 3-day exposure to various gelatin/CS scaffolds. Values are mean ± SD (*n* = 3). No significant difference in the cell viability was observed between the control (without materials) and all the experimental groups (*p* > 0.05); (**b**) Gene expression of IL-6 in RCKs incubated with extract medium conditioned with various gelatin/CS scaffolds for 3 days, measured by real-time RT-PCR. Normalization was done by using GAPDH. Data in the experimental groups are percentages relative to that of control groups (without materials). Values are mean ± SD (*n* = 3). No significant difference in the IL-6 mRNA level was observed between the control and all the experimental groups (*p* > 0.05).

Live/Dead assays have been extensively used in our laboratory to study the cellular response to ophthalmic biomaterials such as gelatin [28], chitosan [29], hyaluronic acid [24], poly(2-hydroxyethyl methacrylate)-*co*-poly(acrylic acid) [30], and gelatin-*g*-poly(*N*-isopropylacrylamide) [10]. Here, the cell viability was quantified by the same method. Figure 6a shows the mean percentage of live cells in the RCK cultures after 3 days of incubation with extract medium conditioned with various gelatin/CS scaffolds. The cell viability in the control, N/E0, N/E0.1, N/E0.2, N/E0.5, and N/E1.0 groups was 99.5% ± 0.5%, 99.1% ± 0.6%, 98.3% ± 0.9%, 99.3% ± 0.6%, 98.8% ± 0.7%, and 98.5% ± 1.0%, respectively. These values did not show a statistically significant difference (*p* > 0.05). The finding of very high viability levels indicates that the exposure to the scaffolds modified at different NHS/EDC molar ratios is not detrimental to corneal keratocyte survival.

The expression level of IL-6 is considered as an indicator associated with biocompatibility assessment [31]. The elevation of IL-6 gene expression level usually reflects the induction of severe foreign body reaction by the test materials. Figure 6b shows the pro-inflammatory gene expression of RCKs exposed to various samples for 3 days. Similar IL-6 expression levels were observed between the control and all the experimental groups (*p* > 0.05), suggesting good cytocompatibility of these gelatin/CS scaffolds. Strehin *et al.* have shown that the injection of the CS-polyethylene glycol scaffold in rats can decrease the inflammatory response when compared to a control polyethylene glycol sample [32]. Although we do not find that the presence of CS alleviates the cellular inflammation, it seems that the modification of gelatin scaffolds with CS does not stimulate pro-inflammatory cytokine IL-6 production by cultured corneal keratocytes.

### 2.7. Protein Adsorption Studies

**Figure 7:**
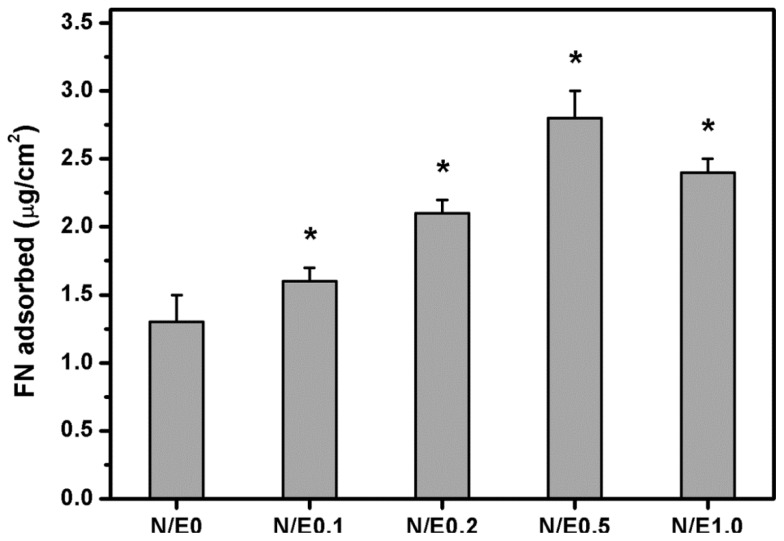
Amount of fibronectin (FN) adsorbed on various gelatin/CS scaffolds as determined by colorimetric assay using a bicinchoninic acid (BCA) protein assay kit. An asterisk indicates statistically significant differences (*p < 0.05; *n* = 4) as compared to the N/E0 groups.

Serum, which contains FN, is an important nutritional component in culture medium [33]. Given that FN is one of the matrix molecules for the mediation of cell adhesion, the investigation of the behavior of FN adsorption is of special interest in connection with RCK adhesion. Figure 7 shows the results of amount of FN adsorbed on various gelatin/CS scaffolds. In the N/E0 groups, the adsorbed FN amount was 1.3 ± 0.2 μg/cm^2^. It was significantly lower than those of the N/E0.1 (1.6 ± 0.1 μg/cm^2^), N/E0.2 (2.1 ± 0.1 μg/cm^2^), and N/E0.5 (2.8 ± 0.2 μg/cm^2^) groups (*p* < 0.05). In addition, it was noted that the adsorbed FN amount was significantly reduced to 2.4 ± 0.1 μg/cm^2^ in the N/E1.0 groups compared to that in the N/E0.5 groups (*p* < 0.05). Our findings suggest that the variation of FN adsorption amount is in accordance with the results of covalent incorporation of CS into the gelatin scaffolds. Wasylnka *et al.* have reported that the negatively charged carbohydrates such as dextran sulfate and heparin tend to bind to the GAG binding domain of FN [34]. This may explain the samples having the highest CS content that shows the largest amount of adsorbed FN among all the scaffolds studied.

### 2.8. Cell Adhesion Assays

**Figure 8:**
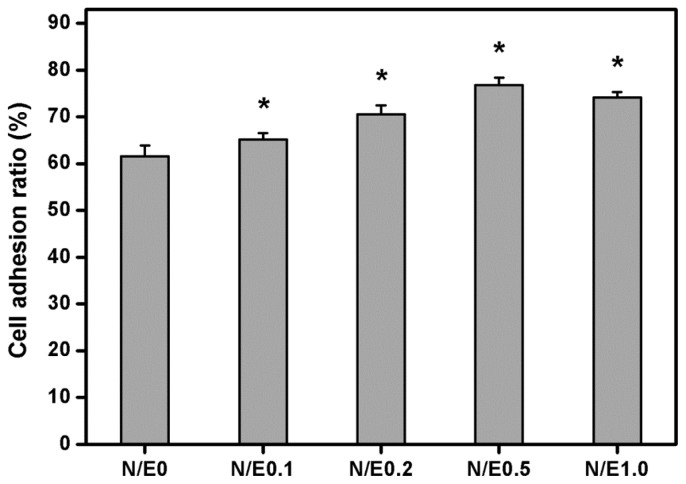
Cell adhesion ratio on various gelatin/CS scaffolds after RCK seeding for 8 h. An asterisk indicates statistically significant differences (*p < 0.05; *n* = 4) as compared to the N/E0 groups.

Attachment of cells to their underlying substratum is essential to achieve successful corneal keratocyte cultures. We have recently shown that even in the absence of serum, the cultivated RCKs adhere well on tissue culture polystyrene plates following incubation with culture medium for 8 h [35]. Therefore, in this study, the adhesion of RCKs to various gelatin/CS scaffolds was quantified by counting cell numbers at the indicated time (Figure 8). The cell adhesion ratio in the N/E0, N/E0.1, N/E0.2, N/E0.5, and N/E1.0 groups was 61.6% ± 2.3%, 65.1% ± 1.4%, 70.5% ± 2.0%, 76.8% ± 1.6%, and 74.1% ± 1.2%, respectively. There were significant differences among these groups (*p* < 0.05). The results indicate that the cell adhesion on CS-modified scaffolds follows the same trend as FN adsorption. Rammelt *et al.* have reported that when compared to the titanium discs coated with type I collagen only, the counterparts treated with CS and collagen are more effective at increasing the cultured osteoblast adhesion [36]. The present data are compatible with their findings, and further suggest that the CS content in the gelatin scaffold plays an important role in the attachment of corneal keratocytes to the biomaterial substrates. Although the mechanism of CS function in RCK adhesion remains unclear, CS is thought to be involved in specific integrin-mediated fibroblast adhesion and the enhancement of focal adhesion kinase phosphorylation during wound healing signaling events [37]. Our future interest will be toward more fully exploring the relationship between the CS molecule and corneal keratocyte adhesion.

### 2.9. Cell Proliferation Assays

**Figure 9:**
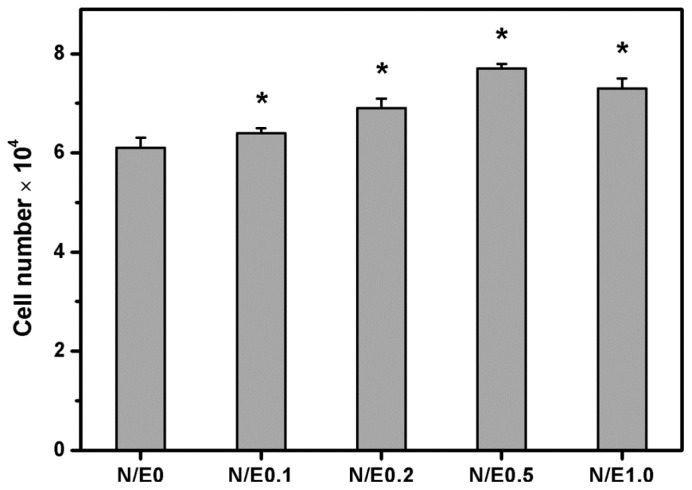
Total cell number on various gelatin/CS scaffolds after RCK seeding for 5 days. An asterisk indicates statistically significant differences (*p < 0.05; *n* = 4) as compared to the N/E0 groups.

CS is an attractive natural-origin polymer that can interact with various growth-active molecules [18]. Milev *et al.* found that the CS promoted cell proliferation mainly due to the ability of this GAG to bind to fibroblast growth factor-2 and potentiate its mitogenic activity [38]. Additionally, Smith *et al.* examined the role of perlecan containing CS chains as a sink for fibroblast growth factor-2 in the growth plate matrix [39]. Figure 9 shows the results of quantitative analysis for RCK proliferation within various gelatin/CS scaffolds modified at different NHS/EDC molar ratios. After 5 days of cultivation, the total cell number in the N/E0 groups was 61,000 ± 2000, which was significantly higher than the initial adherent cell number (*i.e.*, 35000) (*p* < 0.05). In the range of 0.1–0.5, the cell number significantly increased with increasing CS content, thereby with NHS/EDC molar ratio (*p* < 0.05). It was also noted that the number of RCKs was significantly reduced to 73000 ± 2000 in the N/E1.0 groups compared to 77,000 ± 1000 in the N/E0.5 groups (*p* < 0.05), which possibly suggests that the cell proliferation was inhibited after seeding in a three-dimensional culture onto the scaffolds modified at the maximum NHS/EDC molar ratio tested. One potential explanation for this phenomenon is that the cell growth strongly depends on the incorporated amount of CS in the gelatin scaffold. The influence of CS on the cell proliferation has been previously studied. An earlier report from van Susante *et al.* showed that as compared to type I collagen scaffolds, the collagen-CS matrices could significantly enhance the proliferative capacity of chondrocytes [40]. More recently, our group also demonstrated that the covalent linkage of CS to gelatin scaffolds was able to stimulate the RCK proliferation [11]. Here, the CS content in the biopolymer matrices affected by the NHS to EDC molar ratio is confirmed to be critical for regulating corneal stromal cell growth on gelatin/CS scaffolds.

### 2.10. Extracellular Matrix Production Assays

**Figure 10:**
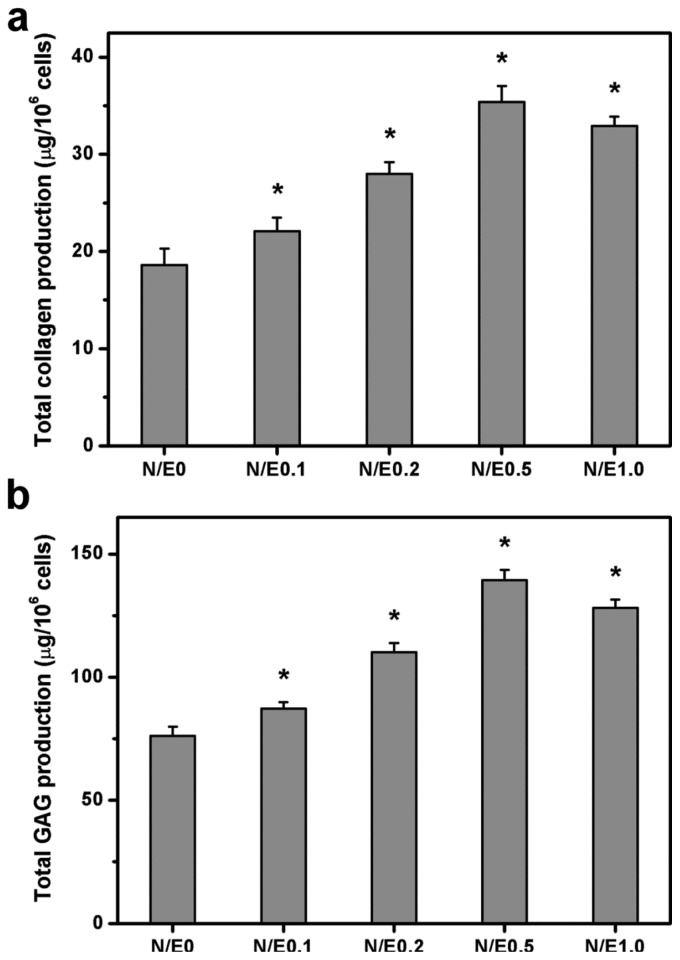
ECM production capacity of RCKs at 5 days after plating on various gelatin/CS scaffolds. (**a**) Collagen content; (**b**) glycosaminoglycan (GAG) content. An asterisk indicates statistically significant differences (* *p* < 0.05; *n* = 4) as compared to the N/E0 groups.

The ECM production capacity of RCKs at 5 days after cultivation on various gelatin/CS scaffolds were examined by measuring the collagen and GAG contents in the supernatants. The results of biochemical assays are presented in Figure 10. The amount of total collagen in the N/E0, N/E0.1, N/E0.2, N/E0.5, and N/E1.0 groups was 18.6 ± 1.7, 22.1 ± 1.4, 28.0 ± 1.2, 35.4 ± 1.6, and 32.9 ± 1.0 μg/10^6^ cells, respectively (Figure 10a). There were significant differences among these groups (*p* < 0.05). In addition, similar variation in the amount of total GAG was observed for the scaffolds modified at different NHS/EDC molar ratios (Figure 10b).

Particularly, the ECM production reached the highest level on the samples from N/E0.5 groups, respectively, with 190% and 183% amplification in collagen and GAG secretion over the counterparts from N/E0 groups. Bassleer *et al.* have shown that CS is able to increase matrix component production by human articular chondrocytes [41]. Cao *et al.* have also demonstrated that the collagen-CS scaffold contains more newly synthesized GAG than in the absence of CS after 2 weeks of chondrocyte culture [42]. In our study, significant differences in the biosynthetic behavior of the corneal stromal cells in the different scaffold materials were found, suggesting the potential role of NHS/EDC molar ratio in governing the CS content in the gelatin matrices for the modulation of biological function in cultured cells.

## 3. Experimental Section

### 3.1. Materials

Gelatin (type A, from porcine skin, 300 Bloom), chondroitin-4-sulfate (CS; molecular weight 20,000), 1-ethyl-3-(3-dimethyl aminopropyl) carbodiimide hydrochloride (EDC), glucose, glucose assay kit (glucose oxidase/peroxidase reagent and *o*-dianisidine reagent), collagenase type I, human plasma fibronectin (FN), bovine serum albumin (BSA), chloramine-T reagent, and Ehrlich’s aldehyde reagent were purchased from Sigma-Aldrich (St. Louis, MO, USA). *N*-hydroxysuccinimide (NHS) was supplied by Acros Organics (Geel, Belgium). Deionized water used was purified with a Milli-Q system (Millipore, Bedford, MA, USA). Dimethylmethylene blue (DMMB) was purchased from Serva Feinbiochemica GmbH (Heidelberg, Germany). Balanced salt solution (BSS, pH 7.4) was obtained from Alcon Laboratories (Fort Worth, TX, USA). Phosphate-buffered saline (PBS, pH 7.4) was acquired from Biochrom (Berlin, Germany). Dispase type II was obtained from Roche (Indianapolis, IN, USA). Medium 199, gentamicin, 1:1 mixture of Dulbecco’s modified Eagle’s medium and Ham’s F-12 medium (DMEM/F12), trypsin-ethylenediaminetetraacetic acid (EDTA), and TRIzol reagent were purchased from Gibco-BRL (Grand Island, NY, USA). Fetal bovine serum (FBS) and the antibiotic/antimycotic (A/A) solution (10,000 U/mL penicillin, 10 mg/mL streptomycin and 25 μg/mL amphotericin B) were obtained from Biological Industries (Kibbutz Beit Haemek, Israel). The bicinchoninic acid (BCA) protein assay kit (Cat. No. 23225) was purchased from Pierce Chemical (Rockford, IL, USA). All the other chemicals were of reagent grade and used as received without further purification.

### 3.2. Modification of Porous Gelatin Scaffolds with Chondroitin Sulfate

The cross-linked porous gelatin membranes were prepared according to the previously published method [7]. Briefly, an aqueous solution of 10 wt% gelatin was obtained by dissolution of gelatin powder in deionized water at 40 °C. The resulting solution was then poured into a polystyrene planar mold (5 × 5 cm^2^, 1.5 cm depth), subjected to freezing at −80 °C for 24 h, and lyophilized at −55 °C for 2 days. All the prepared gelatin sheets were cross-linked by directly immersing the samples in an ethanol/water mixture (8:2, *v*/*v*, pH 4.75) containing 50 mmol/L EDC and 10 mmol/L NHS. The cross-linking reaction was allowed to proceed at 25 °C for 12 h, and the treated samples were thoroughly washed with deionized water to remove excess EDC and urea by-product. The gelatin membrane scaffolds (approximately 100 μm in thickness) were obtained following drying *in vacuo*.

The cross-linking index determined by the ninhydrin assays was 75.4% ± 1.8% [11]. By means of scanning electron microscopic observations and solvent displacement studies of the cross-linked scaffolds, the pore size and porosity was approximately 50 μm and 25%, respectively [11]. The cross-linked scaffold samples (10 mg) were again immersed in an ethanol/water mixture (8:2, *v*/*v*, pH 4.75) containing 10 mmol/L EDC, 0–10 mmol/L NHS, and 0.25% (*w*/*v*) CS. The molar ratio of EDC to COOH of CS was fixed at 1:0.55. The reaction proceeded for 24 h under gentle shaking. To remove ionically bound CS, the treated samples were further washed with 500 mM NaCl, followed by washing with deionized water. In this study, the gelatin/CS scaffolds modified at a NHS/EDC molar ratio of 0.1 was designated as N/E0.1.

### 3.3. Fourier Transform Infrared (FTIR) Spectroscopy

The Fourier transform infrared (FTIR) spectroscopy of various samples was performed using a FT-730 ATR-FTIR Spectrophotometer (Horiba, Japan) according to the previously published method [43]. The spectra were recorded between 3700 and 900 cm^−1^ with a resolution of 8 cm^−1^. The data were analyzed using FTIR spectrum software (Horiba) to obtain quantitative peak information. The results were the average of three independent experiments.

### 3.4. Dimethylmethylene Blue Assays

The CS content of various modified gelatin scaffolds was determined by DMMB assay. After hydrolysis of membranes with 6 N HCl at 105 °C for 6 h, the samples were mixed with DMMB reagent solution (40 mM NaCl; 40 mM glycine; 46 μM DMMB, pH 3.0) [11]. The absorbance was read at 525 nm by using the Multiskan Spectrum Microplate Spectrophotometer (ThermoLabsystems, Vantaa, Finland), and referenced to a standard curve of CS over a range of concentrations from 0.01 to 2.5 nmol/mL. Results were averaged on five independent runs.

### 3.5. Water Content Measurements

For water content measurements, the samples were first dried to constant weight (*W*_i_) *in vacuo* and were immersed in BSS at 34 °C (physiological temperature of the cornea) with reciprocal shaking (50 rpm) in a thermostatically controlled water bath. After 4 h, the swollen membrane scaffolds were weighed (*W*_s_), and the equilibrium water content (%) of the test sample was defined by ((*W*_s_ − *W*_i_)/*W*_s_) × 100 as described previously [44]. Results were averaged on five independent runs.

### 3.6. Mechanical Tests

The uniaxial tensile tests were used to characterize the mechanical properties of various membrane scaffolds [45]. Each sample was immersed in BSS for 4 h to reach the fully swollen state. Subsequently, dumbbell-shaped specimens were prepared by cutting wet membranes under pressure with a suitable mold. The gauge length of the specimens was 10 mm and the width was 5 mm. All measurements were performed at 25 °C and a relative humidity of 50% using an Instron Mini 44 universal testing machine (Canton, MA, USA). The crosshead speed was set at 1 mm/min. Young’s modulus was determined from load-displacement curves. Results were averaged on nine independent runs.

### 3.7. Glucose Permeation Studies

Glucose permeation studies were performed at 34 °C using a horizontal glass diffusion cell (PermeGear, Hellertown, PA, USA) having two stirred chambers with sampling ports. The donor chamber was filled with a 50 mg/mL glucose solution in BSS (3 mL) and receptor chamber with BSS (3 mL). After immersion in BSS until equilibrium swelling, the membranes were placed between the two chambers. During the measurements, all solutions were stirred continuously to provide uniform solute distribution and to reduce boundary layer of glucose. After 12 h, the receptor chamber was sampled and analyzed using a glucose assay kit [46]. Photometric readings at 540 nm were measured with a spectrophotometer (Thermo Scientific, Waltham, MA, USA) and compared with a standard curve of known glucose concentrations. Results were averaged on five independent runs.

### 3.8. Isolation and Culture of Rabbit Corneal Keratocytes

Sixteen adult male New Zealand white rabbits (National Laboratory Animal Breeding and Research Center, Taipei, Taiwan, ROC) weighing 2.5–3.0 kg were used for this study. All animal procedures were approved by the Institutional Review Board and were performed in accordance with the ARVO Statement for the Use of Animals in Ophthalmic and Vision Research. For the isolation of stromal keratocytes, each cornea was exposed to Medium 199 and 50 μg/mL of gentamicin. Under a dissecting microscope (Leica, Wetzlar, Germany), Descemet’s membrane with the attached endothelium was aseptically stripped from the stroma [47]. Then, the remaining stroma with epithelium was incubated in 5 mg/mL of dispase II at 4 °C overnight [35]. Loose epithelial sheets were removed, and stromal discs were cut into small pieces and digested using 4 mg/mL collagenase I for 24 h at 37 °C. The keratocytes were mechanically dissociated into single cells, and a cell pellet was collected via centrifugation (1000 rpm, 25 °C, 5 min). Thereafter, rabbit corneal keratocytes (RCKs) were resuspended and maintained in regular culture medium consisting of DMEM/F12, 10% FBS, and 1% A/A solution. Cultures were incubated in a humidified atmosphere of 5% CO_2_ at 37 °C. Medium was changed every other day. Confluent cell layers were subcultured by treating with trypsin-EDTA for 2 min and seeded at a 1:4 split ratio. Only third-passage RCKs were used for this study.

### 3.9. *In Vitro* Biocompatibility Studies

The *in vitro* biocompatibility evaluation of the membrane scaffolds was conducted in adaptation from the ISO10993-5 standard test method [30]. The material samples were sterilized in a 70% ethanol solution overnight and thoroughly rinsed in sterilized PBS. A single extract of the test article was prepared using regular growth medium containing DMEM/F12, 10% FBS, and 1% *A*/*A* solution. The extracts were obtained by incubation of the sterilized materials with culture medium at 37 °C for 24 h with an extraction ratio of 0.2 g/mL. Each test extract was then placed onto RCK cultures with a seeding density of 5 × 10^4^ cells/well, and maintained at 37 °C in the presence of 5% CO_2_ for 3 days. The cells in regular growth medium without contacting material samples served as control groups.

Cell viability was determined using the Live/Dead Viability/Cytotoxicity Kit from Molecular Probes (Eugene, OR, USA) [29]. This assay uses intracellular esterase activity to identify the living cells; the process cleaves the calcein acetoxymethyl to produce a green fluorescence. Ethidium homodimer-1 can easily pass through the damaged cell membranes of dead cells to bind to the nucleic acids, yielding a red fluorescence. After washing three times with PBS, the cultures were stained with a working solution consisting of 2 μL of ethidium homodimer-1, 1 mL of PBS, and 0.5 μL of calcein acetoxymethyl. Under fluorescence microscopy (Axiovert 200M; Carl Zeiss, Oberkochen, Germany), three different areas each containing approximately 500 cells were counted at 100× magnification. All experiments were performed in triplicate, and the viability of the RCK cultures was expressed as the average ratio of live cells to the total number of cells in these nine different areas.

Pro-inflammatory cytokine interleukin-6 (IL-6) expression was detected at messenger RNA (mRNA) levels [48]. Total RNA was isolated from cells with TRIzol reagent according to the manufacturer’s procedure. Reverse transcription of the extracted RNA (1 μg) was performed using ImProm-II (Promega, Madison, WI, USA) and Oligo(dT)_15_ primers (Promega). The primers used to amplify the rabbit IL-6 complementary DNA (cDNA) were 5′-AAGAAAACACCAGGGTCAGCAT-3′ (sense) and 5′-CTTGAGGGTGGCTTCTTCATTC-3′ (antisense), and those used to amplify the internal control cDNA, glyceraldehyde-3-phosphate dehydrogenase (GAPDH), were 5′-TTGCCCTC AATGACCACTTTG-3′ (sense) and 5′-TTACTCCTTGGAGGCCATGTG-3′ (antisense). Quantitative real-time reverse transcription polymerase chain reaction (RT-PCR) was performed on a Light-Cycler instrument (Roche Diagnostics) according to the manufacturer’s instructions with FastStart DNA Master SYBR Green I reagent (Roche Diagnostics). Each sample was determined in triplicate and the results for IL-6 were normalized to the level of GAPDH mRNA.

### 3.10. Protein Adsorption Studies

The human plasma FN solution (20 μg/mL) was poured into each well of a 24-well plate coated with various membrane samples with an area of 2 cm^2^. After 1 h of incubation at 37 °C, each well was rinsed with PBS three times. The adsorbed proteins were desorbed by soaking in 1% SDS for 30 min at room temperature. Subsequently, the protein concentrations were determined by a BCA protein assay kit using BSA as the standard [8]. All the experiments were run in quadruplicate.

### 3.11. Cell Adhesion Assays

The RCKs (5 × 10^4^ cells/well) were seeded into each well of a 24-well plate coated with various membrane samples with an area of 2 cm^2^. After 8 h of incubation at 37 °C, the wells were washed three times with PBS to remove non-attached cells, and the number of RCKs attached to the sample surface was determined by the CellTiter 96 Aqueous Non-Radioactive Cell Proliferation MTS Assay (Promega), in which tetrazolium compound (3-(4,5-dimethylthiazol-2-yl)-5-(3-carboxymethoxyphenyl)-2-(4-sulfophenyl)-2H-tetrazolium, inner salt; MTS) is bio-reduced by cells to form a water-soluble colored formazan [23]. The amount of colored product is proportional to the number of metabolically active cells. 100 μL of the combined MTS/phenazine methosulfate (PMS) (20:1) reagent was added to each well of the 24-well plate, and incubated for 3 h at 37 °C in a CO_2_ incubator. The data of absorbance readings at 490 nm were measured using the Multiskan Spectrum Microplate Spectrophotometer (ThermoLabsystems), and referenced to a standard curve of absorbance *versus* cell number as determined by hemacytometer. All experiments were performed in quadruplicate. The cell adhesion ratio was expressed as the average ratio of number of attached RCKs to the total number of plating cells (*i.e.*, 5 × 10^4^ cells).

### 3.12. Cell Proliferation Assays

After attachment of RCKs (3.5 × 10^4^ cells/scaffold) onto the sterilized membrane scaffolds, the samples were washed three times with PBS to remove non-attached cells. The cell-polymer constructs were immediately transferred to a new culture well, and the medium was replaced with fresh growth medium. After 5 days of incubation at 37 °C, the proliferation of the cells was evaluated using the CellTiter 96 Aqueous Non-Radioactive Cell Proliferation MTS Assay, as described in Section 3.11. In this study, in order to avoid the possible interference of cell migration and growth onto the bottom of the well, the cell-polymer constructs were again transferred to another new culture well before the MTS assay was performed. All experiments were carried out in quadruplicate.

### 3.13. Extracellular Matrix Production Assays

After RCK cultivation on various scaffolds for 5 days, the supernatants were respectively collected for analyses of collagen and glycosaminoglycan (GAG) contents. Blank experiments (culture medium exposed to the cell-free scaffolds) were conducted simultaneously for correction of results. The amount of hydroxyproline, which is an amino acid marker of collagen, was determined with some modifications of the protocol used for quantification of hydroxyproline in aqueous humor [7]. Briefly, after hydrolysis with 6 N HCl for 18 h at 110 °C, the samples were mixed with a buffered chloramine-T reagent, and the oxidation was allowed to proceed for 25 min at room temperature. After the addition of Ehrlich’s aldehyde reagent to each sample, the absorbance was read at 550 nm by using the Multiskan Spectrum Microplate Spectrophotometer (ThermoLabsystems) and compared with a standard calibration curve to quantify the amount of hydroxyproline. The GAG content was determined by colorimetric assay using DMMB reagent, as described in Section 3.4. All experiments were conducted in quadruplicate.

### 3.14. Statistical Analysis

Results were expressed as mean ± SD. Comparative studies of means were performed using one-way analysis of variance (ANOVA). Significance was accepted with *p* < 0.05.

## 4. Conclusions

For the development of corneal stromal tissue engineering, it is very important to optimize the fabrication of cell scaffolds. In the present work, the porous gelatin membranes were modified with CS by means of carbodiimide chemistry. With the optimum NHS to EDC molar ratio of 0.5, chemical treatment can achieve relatively high CS content in the gelatin scaffolds, thereby enhancing the water content, glucose permeation, FN adsorption, and keratocyte adhesion. Although all the test samples have good cytocompatibility, the materials with highest CS content show the greatest stimulatory effect on the proliferation and biosynthetic activity of corneal stromal cells, indicating that these biopolymer matrices exhibit better performance than those modified at NHS/EDC molar ratio of 0.2 (*i.e.*, the previously optimized condition). The findings suggest that the NHS/EDC molar ratio used for biopolymer modification may play a crucial role in the characteristics and biofunctionalities of gelatin/CS scaffolds.

## Supplementary Information
