# Supplementary material for: Corneal Stromal Cell Growth on Gelatin/Chondroitin Sulfate Scaffolds Modified at Different NHS/EDC Molar Ratios
Source: Int J Mol Sci. 2013 Jan 21;14(1):2036–55. doi: 10.3390/ijms14012036 (PMC3565364; doi:10.3390/ijms14012036)
Supplement: Supplementary file 1 [file ijms-14-02036-s001.pdf]

Supplementary Information

Figure S1. FTIR spectra of the gelatin/CS scaffolds modified at different NHS/EDC molar ratios.

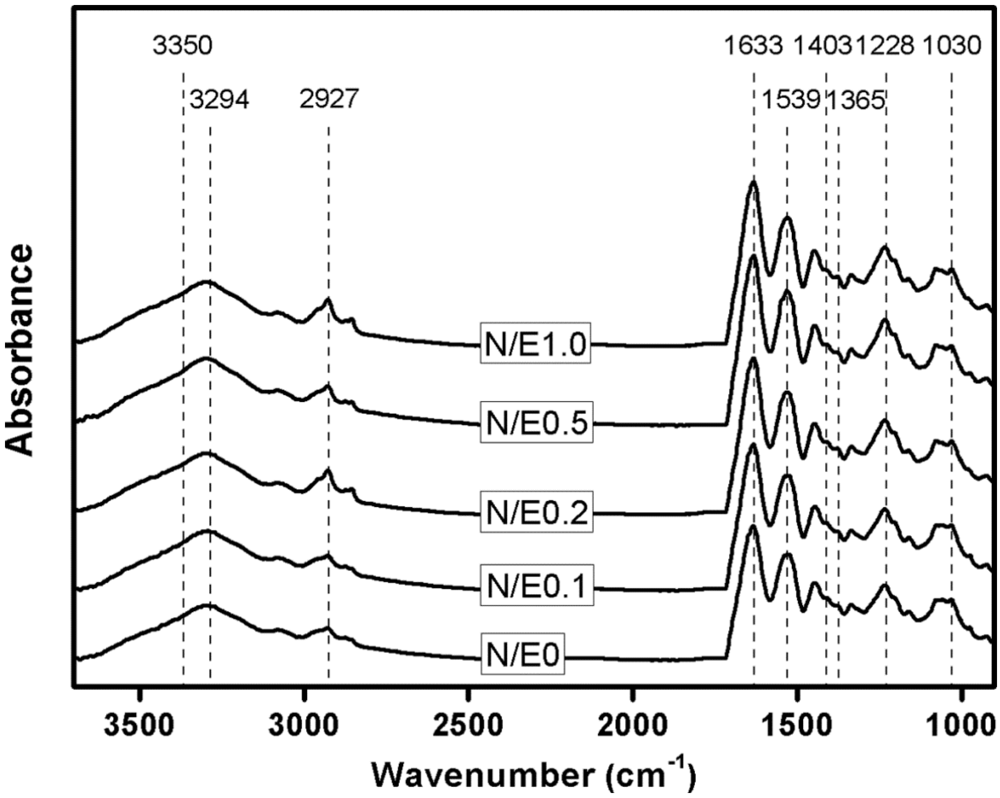

© 2013 by the authors; licensee MDPI, Basel, Switzerland. This article is an open access article distributed under the terms and conditions of the Creative Commons Attribution license (http://creativecommons.org/licenses/by/3.0/).
